# Reformulation of Tunisian Sun-Dried Merguez with Camel Meat: Characterization of Physicochemical and Compositional Changes in Organic Acids, Fatty Acids, Volatile Compounds, and Minerals

**DOI:** 10.3390/foods13071032

**Published:** 2024-03-28

**Authors:** Khaoula Belguith, Zeineb Jrad, Olfa Oussaief, Mohamed Debara, Talel Bouhemda, Haifa Sebii, Mohamed Hammadi, Halima El Hatmi

**Affiliations:** 1 Physiopathology, Food and Biomolecules Laboratory (LR17ES03), Higher Institute of Biotechnology Sidi Thabet, University of Manouba, Ariana 2020, Tunisia; 2 Livestock and Wildlife Laboratory (LR16IRA04), Institute of Arid Land, University of Gabes, Medenine 4100, Tunisiamhammadi70@gmail.com (M.H.); halima.elhatmi@ira.rnrt.tn (H.E.H.); 3 Central Laboratory, Institute of Arid Land, University of Gabes, Medenine 4100, Tunisia; mohamed.dbara@gmail.com (M.D.);; 4 Laboratory of Analysis Valorization and Food Safety, National Engineering School of Sfax, University of Sfax, Sfax 3038, Tunisia

**Keywords:** merguez, sun drying, camel meat, hump fat, lactic acid, oleic acid

## Abstract

Traditional sun-dried merguez is an authentic Tunisian dried sausage made with a large number of spices and herbs, which was reformulated in this study with camel meat and hump fat and dried as in the artisanal process. This research studied the physicochemical, microbiological, and chemical compositional changes that occurred in fresh camel merguez (FCM) after 12 days of drying to achieve traditional dried camel merguez (DCM). The results showed significant weight loss (54.1%), as well as significant decreases in pH (5.20–4.97), moisture (60.5–12.3%), and water activity (0.986–0.673). These results and the acceptable microbiological quality of DCM can explain the safety of traditionally practiced long-term storage at room temperature. All chemical compositions increased upon drying. The composition of DCM included several organic acids, mainly lactate (2820 mg.kg^−1^); diverse unsaturated fatty acids, in particular oleic acid (33.2%); and various minerals, specifically iron (8 mg per 100 g), in addition to volatile compounds impacted by herbs and spices rich in terpenes (56.3%). These results can be useful for investing in indigenous products and promoting the exploitation of camel meat.

## 1. Introduction

Merguez is a known traditional spicy sausage from Tunisia and all of North Africa [1], widely consumed in different countries, including Europe [2]. Traditional sun-dried merguez, an authentic Tunisian dry sausage, is well known in Tunisia but rarely described in the literature [3,4]. It is made from sheep tail fat and beef or lamb meat, seasoned abundantly with salt, herbs, and spices and stuffed into natural sheep casings, and then dried in the open air in a clean place and stored in an olive oil-filled pottery recipient as El-Guedid [4,5]. It is used like EL-Guedid as a flavoring ingredient to prepare various Tunisian dishes such as couscous and is not consumed raw [6]. It has a characteristic flavor and color improved by harissa, a hallmark of Tunisian food. Harissa is a paste of hot chili peppers spiced with garlic, caraway, and coriander [3]. In addition to mint and fennel, these ingredients provide an essential source of aromatic and bioactive healthy compounds in dry merguez. Flores and Piornos [7] presumed that in addition to being part of an ethnic culinary heritage, the popularity of traditional products relates to their natural composition and sensory and nutritional quality.

Among the culinary heritage of North African countries, camel meat is used as an alternative to beef, given the abundance of camels in arid areas. But, despite the extensive distribution of camelids and the vital capacity of the animal to adapt to climate changes, the exploitation of camel meat has not yet been fully explored [8,9].

Camels can provide a source of low-cost and high-quality meat, rich in minerals and characterized by a higher protein yield, lower fat and cholesterol content, lower saturated fatty acid content, and higher monounsaturated fatty acid content than veal [10,11].

Only a few studies have reported on camel meat products, specifically camel meat sausages [12,13,14,15]. All of them were considered dry-fermented camel meat sausages. The process of making dry-fermented sausages includes two controlled steps: a first fermentation step with high relative humidity (85–90%) and a temperature of more than 20 °C, followed by a second ripening step with a lower temperature (≤20 °C) and lower relative humidity (75–80%). Conversely, sun drying is a primitive process which takes about 8 to 12 days, with uncontrolled temperature and humidity. According to the classification of Gagaoua and collaborators [1] and based on the traditional preparation method, dried merguez can be considered as El-Guedid, a dried, not fermented, meat product. The uncontrolled drying conditions remain a safety-limiting factor for commercialization at a broader level, as is the case for most artisanal and traditional products [16]. 

In this context and considering the scarcity of Tunisian traditional meat product studies, the current study aims to characterize dried camel merguez (DCM) and determine the changes to fresh camel merguez (FCM) that occur during the drying process in terms of the physicochemical, microbiological, and compositional properties of organic acid, fatty acids, and volatile compounds. To our knowledge, this is the first study of authentic Tunisian dried camel merguez as manufactured in the Tunisian tradition, which will reveal helpful information for the development of and improvement in camel meat products and contribute to preserving the national cultural heritage.

## 2. Materials and Methods

### 2.1. Materials and Chemicals

Camel meat from biceps femoris muscles and hump fat was purchased from a local market and came from three different animal carcasses. The spices (from Epices et Saveurs, Piments Chakroun Chams S.A.R.L., Sfax, Tunisia) were ground hot red chili peppers and “tabeul”. The latter is a Tunisian spice mixture containing approximately 70% coriander, 20% caraway, and 10% dried garlic. Powdered fennel seeds and ground dried mint were obtained from Kamy S.A. (Nabeul, Tunisia). Harissa was purchased from ETS Zgolli S.A.R.L. (Nabeul, Tunisia). The chemical products used in the analysis were acquired from Sigma-Aldrich (St. Louis, MO, USA).

### 2.2. Formulation and Manufacturing of Dry Merguez

Fresh camel merguez (FCM) was formulated with camel meat and hump fat (Table 1) in three independent replicate batches (2 kg/batch). Each batch was made from a single camel carcass. Initially, the fat and meat were sliced (50 mm) and ground using a meat mincer with a 6 mm plate hole size (Fimar, Model 22RS, Villa Veruccino, Italy). The ingredients were added into refrigerated potable water (0.083 L) and then manually mixed well with ground meat. The resulting meat emulsion was stuffed into natural sheep casings using manual sausage fillers (Fimar, Model LT70R, Villa Veruccino, Italy). The three batches were drained by hanging them in a refrigerated room at 4 ± 2 °C for 2 h, and then they were dried in the shade for 12 days to obtain dried camel merguez (DCM). During the drying process, every 48 h, each batch was weighed, and a sample of approximately 10 g was taken for immediate water activity (a_w_) measurement. The FCM and DCM samples were stored at −20 °C in a sealed brown glass container for 3 to 15 days and thawed in a refrigerator overnight before analysis. 

### 2.3. Environmental Conditions, Drying Weight Loss, Water Activity, and pH

During the drying process, the environmental temperature and relative humidity (RH) were checked twice a day using an air quality detector (DM306D, USA) with infrared (NDIR) and laser-scattering detection sensors. The weight of the merguez was determined every 48 h during the 12 days of drying for the three batches. The relative drying weight loss (RWL) and the daily drying weight loss (DWL) were calculated according to Equations (1) and (2), as follows:

(1) $$\mathrm{RWL}=\left[1-\left(\frac{\mathrm{Wdi}}{\mathrm{Wd}0}\right)\right]\times 100$$

(2) $$\mathrm{DWL}=\;\left[1-\left(\frac{\mathrm{Wdi}+1}{\mathrm{Wdi}}\right)\right]\times 100$$

where Wd_i_ is weight on day i (0 ≤ i ≤ 12).

Water activity (a_w_) was appraised as indicated by ISO 18787 [17] with a NOVASINA^®^aw meter (LabSwift-aw, Novasina AG, Lachen, Switzerland) every 48 h. The pH was assessed for each batch according to ISO 2917 [18] immediately before storage at −20 °C. Briefly, a 1:10 dilution of 10 g of sample in distilled water was measured using a WTW™ pH meter (inoLab™ 7110, Weilheim, Germany) with an electrode (SenTix^®^ 51, Weilheim, Germany), previously calibrated with pH 7, 4.01, and 10.01 buffers (XS Instruments, Via della Meccanica, Carpi, MO, Italy).

### 2.4. Microbiological Analysis 

Microbiological analysis was performed on fresh and final dried products for each batch. First, 25 g of each sample was added to 225 mL of buffered peptone water (Chemosolute 8449, Th. Geyer GmbH & Co. KG, Renningen, Germany) and homogenized. A series of decimal dilutions was then prepared with a tryptone salt broth (BK014HA, Biokar diagnostics, Allonne, France) and inoculated to the media according to the appropriate standard methods: plate count agar (70152, Fluka, Steinheim, Switzerland) for mesophilic aerobic bacteria (ISO 4833-1) [19], de Man–Rogosa–Sharpe agar (CM1153B, Oxoid Ltd, Hampshire, UK) for lactic acid bacteria or LAB (ISO 15214) [20], yeast and mold agar (CM0920B, Oxoid Ltd) for yeast and mold (NF V 08-059) [21], violet red bile agar (CM1082B, Oxoid Ltd) for enterobacteria at 37 °C (NF V 08-054) [22], chromogenic tryptone bile X-glucuronide agar (AGTB-00I-500, Labkem Ltd, Kilbake House, Corballis, IRE) for Escherichia coli β glucuronidase+ (ISO 16649-2) [23], and iron sulfite agar (Condalab Torrejón de Ardoz, Madrid, Spain) for sulfite-reducing anaerobic bacteria (ISO 15213) [24].

The enumeration of positive coagulase staphylococcus was accomplished according to NF V 08 057-1 [25], and *Listeria monocytogenes* was detected as described in ISO 11290-1 [26]. 

### 2.5. Chemical Composition

#### 2.5.1. Moisture, Protein, and Fat Determination

The moisture content was determined using the oven-drying method at 105 °C for 24 h [27]. The total protein was analyzed using the micro-Kjeldahl distillation method via a distilling unit (J. Selecta digestion unit and PRO-NITROII, Barcelona, Spain). The protein content was calculated as N × 6.25. The total fat content for the meat products was determined according to ISO1443 [28] and consisted of boiling samples with hydrochloric acid, filtrating, drying, and extracting using n-hexane solvent.

#### 2.5.2. Organic Acid Analysis

Organic acids were assessed as described by Dursun and collaborators [29] with some modifications. Samples were mixed with sulfuric acid (10 mM) at a ratio of 4:1 (*w*/*v*) and then centrifuged (10,000× *g*, 20 min, 4° C). The supernatant was filtered through a 0.45 μm syringe filter, and 10 μL of the sample was injected into the HPLC system (Shimadzu UFLC XR). Separation was performed using an Agilent Hi-Plex H organic acid column (7.7 × 300 mm, 7.8 μm). The mobile phase was the sulfuric acid solution (100 mM), and the flow rate was 0.6 mL/min at 50 °C. Organic acids were identified at a wavelength of 210 nm, and their proportions were calculated by comparing the retention times and peak areas with standard solutions of each organic acid.

#### 2.5.3. Fatty Acid Identification

Lipids were extracted using hexane/isopropanol solvents [30]. The samples were subjected to methylation using a KOH solution (2 N) prepared in methanol. The mix was thoroughly agitated with hexane to extract the fatty acid methyl esters (FAMEs). The analysis of the FAMEs was performed using gas chromatography (QP2010 Shimadzu Tokyo, Japan) coupled with mass spectrometry. The column used was a fused silica capillary (Supelcowax-TM10, 30 m length × 0.25 mm i.d. and 0.25 µm film thickness, Bellefonte, PA, USA). The injection conditions were 250 °C, a 10.0 split ratio, and a 1.20 mL/min flow rate. The carrier gas used was helium at 99.99% purity. The quantification and identification of fatty acids were estimated using the GC-MS solution program and Wiley 275 mass spectra libraries (software, D.03.00, Palo Alto, CA, USA).

#### 2.5.4. Volatile Compounds

Aroma compounds were analyzed using a QP2010 Shimadzu gas chromatograph with an RTX-5MS capillary column (30 m × 0.25 mm i.d. and 0.25 µm film thickness, Bellefonte, PA, USA) [31]. The electron ionization mass spectra were collected using a mass spectrometer with an ion source temperature of 200 °C and a mass range of 35–500 *m*/*z*. The volatile compounds of different merguez samples were identified by matching the mass spectra to those in the Wiley 275 mass spectra library (software, D.03.00, Palo Alto, CA, USA).

#### 2.5.5. Mineral Analysis

The dry matter was incinerated at 550 °C for 6 h in an electric muffle furnace. Then, 3 mL of a hydrochloric acid solution (1 N) and 3 mL of deionized water were added to the obtained ash, boiled for 5 min, and filtrated before mineral analysis. The minerals (Na, K, Mg, Zn, Ca, and Fe) were measured using atomic absorption spectrophotometry (Shimadzu AA-6800, Shimadzu, Germany), according to ISO6869 [32]. 

### 2.6. Statistical Analysis

All tests were repeated at least three times and were statistically analyzed using XLSTAT (version 2015.5; Addinsoft, Paris, France). The data were checked for normal distribution and homogeneity, and then evaluated using analysis of variance (ANOVA) followed by Tukey’s test to determine significance at the 5% probability level.

## 3. Results and Discussion

### 3.1. Weight Loss, Water Activity, and pH 

**Figure 1:**
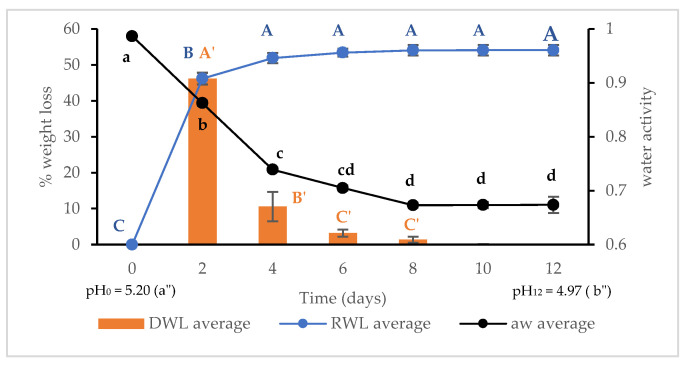
Changes in daily drying weight loss (DWL), relative drying weight loss (RWL), water activity (a_w_), and pH during drying time. Data are presented as the mean ± standard error (S.E.). The same letters (in lowercase for a_w_, uppercase for RWL, and prime uppercase for DWL) indicated for the same parameter over time are not significantly different according to ANOVA (*p* < 0.05).

During drying, the average environmental temperature and relative humidity (RH) were 22 ± 2 °C and 52 ± 6%, respectively. The drying behavior of the traditional process was monitored over time via the RWL and DWL (Figure 1). The RWL curve showed a characteristic drying curve composed of three typical phases [33]. The first phase of significant weight loss (*p* < 0.05) occurred over two days (46.2%) and was mainly caused by the exudation of the free aqueous phase and by the induction of heat from the environment to the surface of the merguez. A second phase (second to fourth day) of deceleration of weight loss, confirmed by a significant decrease (*p* < 0.05) in the DWL and a_w_, was produced by the removal of water from the surface. The third phase of the RWL curve was stable from the fourth day (51.9%) to the eighth day (54.0%). The DWL and a_w_ decreased continually until the eighth day, which was probably caused by the internal moisture diffusion to the surface. After the eighth day, no weight loss occurred, and the a_w_ remained stable at 0.673. Despite obtaining the same trend of the RWL curve compared to dry-fermented sausage, the decrease in weight loss to attain DCM was notable and rapid [12,34]. Likewise, the water activity was very low after drying compared to the reported studies of dry-fermented camel meat sausage [14,15]. The final low a_w_ (0.673) provided evidence for the drastic dehydration of the merguez. This can be explained by the uncontrolled low environmental relative humidity of 52 ± 6% during drying. Based on these results, dried merguez can be distinguished from other types of dry-fermented sausages prepared at higher RH (>70%).

**Figure 2:**
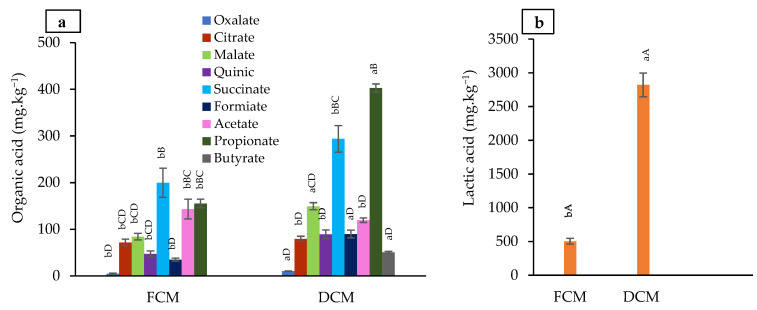
Organic acid concentration (**a**) and lactic acid concentration (**b**) in fresh camel merguez (FCM) and in dried camel merguez (DCM) in mg.kg^−1^. Data are presented as the mean ± S.E. The same letter in lowercase indicates no significant difference according to ANOVA (*p* < 0.05) between FCM and DCM for each organic acid. The same letter in capital letters indicates no significant difference according to ANOVA (*p* < 0.05) between the different organic acid concentrations in FCM or DCM separately.

The pH value of the merguez during drying decreased significantly (*p* < 0.05) from 5.20 to 4.97 (Figure 2). Similar results were observed for dry-fermented camel sausages inoculated with starters [13,15] and in camel sucuk [14]. Camels have a high gluconeogenesis capacity due to their hump; the glucose concentration in camel blood is twice that of other ruminants [35]. The pH of camel meat from the biceps femoris muscle was reported to be 5.94 [36]. Besides the pH of the meat, the heterogeneity of the ingredients, especially chili pepper and harissa, contributed to speeding up the acidification process and decreasing the pH, as deduced in salami with fermented chili powder [37].

### 3.2. Microbiological Change 

The results of the microbiological analysis before and after drying are presented in Table 2. The mesophilic aerobic bacteria, lactic acid bacteria (LAB), *Escherichia coli*, positive coagulase staphylococcus, and *Listeria monocytogenes* results were compared to the limits established by the European Commission Regulation (EC) 2073/2005 [38] and the FCD (version 13/12/2021) [39] relative to the microbiological criteria applicable to meat and products thereof. The fresh camel merguez (FCM) results were compared to raw minced product limits with ingredients such as sausages (intended for cooking). The dried merguez was compared to salted cured raw meat (not intended for cooking), despite dried merguez being cooked in the Tunisian culinary tradition for ethnic reasons. The microbiological results of the fresh and dried merguez met the previous regulations, except for the *Escherichia coli* counts in the FCM, which exceeded these regulations (>2.7 log CFU/g). The inconvenience of *E. coli* has been widely reported. Sallam and collaborators [40] highlighted that camel meat may be a vehicle for multi- and extensively drug-resistant *E. coli*. No *Listeria monocytogenes* were detected, as Bader and collaborators deduced in dry camel meat [5].

Despite the absence of added nitrate and nitrite, commonly used to reduce Clostridium in dry-fermented sausages, the volume of sulfite-reducing anaerobic bacteria was below 10 CFU/g in the final product. However, the isolation of multi-drug-resistant *Clostridium perfringens* and *Cl. difficile* in camel meat in Saudi Arabia requires vigilance in interpreting the results of microbiological analyses [41].

The initial bacterial load of mesophilic bacteria, enterobacteria, yeast, and mold was comparable to previous studies of dry-fermented camel sausages [12,13]. After 12 days of drying, all counted flora decreased in the DCM (Table 2). Similar results were deduced for yeast, mold, and enterobacteria in dry-fermented camel sausages using a starter [13]. On the contrary, the mesophilic aerobic bacteria and LAB increased in these sausages during 14 days of ripening, even without using a starter [12,13,15]. Indeed, their incubation at a controlled temperature (5 days at 24 °C and 23 days at 14 °C) and a higher RH (90–75%) enhanced the growth of LAB and mesophilic aerobic flora.

The LAB count in the FCM was more than 6.0 log CFU·g^−1^, giving rise to the production of lactic acid and organic acid (Figure 2), which decreased the pH to a low level and caused the autoinhibition of or reduction in LAB and all flora in the DCM in synchronization with the critical decrease in a_w_. The same decline was reported for sun-dried camel Gueddid, but this occurred slowly [5]. This is probably due to the difference in texture between ground meat and whole meat pieces. DCM, obtained with low RH (52 ± 6%) and high temperature (22 ± 2 °C) conditions during drying and characterized by a very low aw (0.673) and pH (4.97), matches the classification of sausages (pH < 5 and a_w_ < 0.91) stated by Halagarda and Wójciak as a shelf-stable meat product that does not need refrigeration [42]. The low a_w_ obtained (<0.84) at 20–25 °C was described to be the optimum condition to avoid food-borne pathogens [43] and ochratoxin production [44].

### 3.3. Change in Chemical Composition

#### 3.3.1. Moisture, Protein, and Fat Composition

The merguez composition, including moisture, protein, and fat (Table 3), showed a significant decrease in moisture (*p* < 0.05) and a significant increase in protein and fat in the dried merguez. The critical decline in moisture from 60.5% to 12.3% confirmed the previous finding regarding the drastic dehydration and low a_w_ of DCM. Drying significantly increased the protein and fat composition. Despite the difference in the process, these results were comparable to other reported dry-fermented camel meat sausages [12,13,14].

#### 3.3.2. Organic Acid Composition

The concentrations of ten organic acids (mg·kg^−1^) measured before and after drying are shown in Figure 2. The concentrations of all organic acids increased upon drying. Lactate was significantly (*p* < 0.05) the most dominant organic acid in FCM, and increased significantly to 2820 mg·kg^−1^ in the DCM. A similar concentration was deduced from meat broth containing *Pediococcus pentosaceus*, a promoting strain isolated from dry-fermented sausage with high lactic acid production and antifungal components [45]. 

The dominance of lactate in DCM is proof of intensive LAB fermentation. This probably occurred at the beginning of the drying period when the humidity and a_w_ were optimal. Fermentation may be ensured through the tricarboxylic acid cycle, based on the presence of all organic acids of the tricarboxylic acid cycle, especially citrate. Lactate, propionate, formiate, butyrate, acetate, malate, succinate, and citrate have been widely cited as organic acids generated via homolactic and heterolactic acid bacteria fermentation [46]. The low concentrations of enterobacteria, especially *Escherichia coli* and *Staphylococcus*, and the absence of *Listeria monocytogenes* in the DCM (Table 2) confirmed that lactic acid remains the principal anti-spoilage acid [47], inducing a local microenvironment unfavorable to pathogens [48]. Likewise, the decrease in yeast and mold confirms the antifungal capacity of lactic acid described in many fermented and traditional foods [45]. Besides lactic acid, propionate was the next dominant organic acid in the DCM. The adjustment of a selective medium for enumerating LAB in food with propionic acid at low pH (5.2–6.2) was reported to inhibit the growth of all indicator microorganisms. Still, it enhanced the growth of LAB under anaerobic conditions [49]. LAB play the role of a bio-preservative in meat products to attain a longer shelf life [50]. In addition to their acidifying capacity and antimicrobial and antifungal activity, the produced organic acids are used as flavorings in various processed foods [46]. 

#### 3.3.3. Fatty Acids

The fatty acid composition (g/100 g of total fatty acids) of the dried merguez was analyzed and compared to fresh hump fat to determine the contribution of the latter to the composition of the fatty acids in the sausage. Table 4 shows that the hump fat contained more saturated fatty acids (60.5%) than the DCM (58.3%), and, inversely, the DCM had more unsaturated fatty acids (41.3%) than the hump fat (38.7%). Similar results were deduced by comparing camel meat and hump fat [11]. Nine saturated and six unsaturated components were distinguished in the DCM. Only tridecanoic acid C13:0 was not detected in the hump fat, and arachidonic acid C20:4 (*n*-6) was not detected in the DCM. Previous studies have detected arachidonic acid C20:4 (*n*-6) and tridecanoic acid C13:0 in camel meat, hump fat, and sucuk [11,14,51]. Such a difference can be attributed to the dissimilarity in camel strains from Asia (*Camelus bacterianus*) and North Africa (*Camelus dromedarius*). Oleic acid was significantly the most dominant fatty acid in the DCM (33.2%) and in fresh hump fat (32.6%). Camel meat had a higher content of oleic acid than hump fat [11]. Oleic acid is the primary healthy acid in the Mediterranean diet [51]. The diversity of unsaturated fatty acids (C14:1, C16:1, C17:1, C18:1 (*n*-9), C18:2 (*n*-6), and C20:1) in DCM promotes the nutritional value of this traditional food. Despite the significant proportion of palmitic and stearic acids in the DCM, the saturated fatty acid concentrations were reported to be significantly lower in camel meat than beef, mutton, and poultry [52]. 

#### 3.3.4. Volatile Compounds

**Figure 3:**
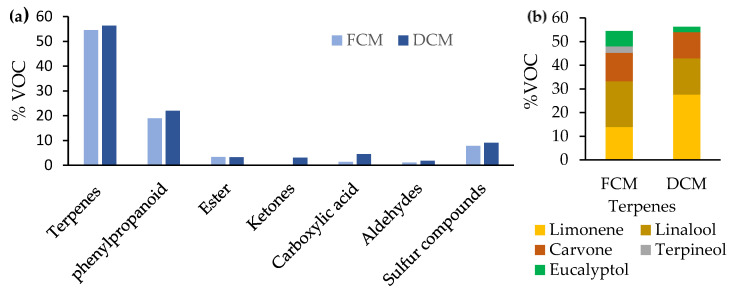
(**a**) Volatile compounds (%) in fresh camel merguez (FCM) and dried camel merguez (DCM). (**b**) Composition of volatile compounds of terpenes and derivates.

Using gas chromatography, 25 volatile compounds were detected (not shown) and classified according to their functional groups in terpenes and derivates, phenylpropanoids, esters, ketones, carboxylic acids, aldehydes, and sulfur compounds (Figure 3). The most detected volatile compounds were monoterpenes and derivatives in both the FCM (54.5%) and DCM (56.3%). Compared to the dry-fermented camel sausage “sucuk”, which contained 46 volatile compounds and 62% terpenes [14], DCM seemed less flavored. The most abundant component of terpenes in the DCM was limonene (27.7%). Monoterpenoid diversity in merguez probably came from the added spices, herbs, and chili pepper. Borrajo and collaborators deduced that no terpenoids were detected in dry-fermented sausages without spices and herbs [53]. The different volatile compounds detected are characteristic of the merguez seasoning, such as limonene (a monocyclic monoterpene) and carvone (an oxygenated monoterpene) that exist in chili pepper [54], mint [55], coriander, and caraway [56], in addition to the distinctive volatile compounds of fennel, like linalool (an acyclic monoterpenoid), terpineol (a cyclic monoterpenoid), eucalyptol, fenchone (two bicyclic monoterpenoids), estragole (a phenylpropanoid), and limonene [57]. The above volatile compounds are widely labeled as structural constituents of different essential oils and their main bioactive healthy compounds [58]. 

Regarding their high concentration and low odor threshold [59], terpenes, followed by sulfur compounds (diallyl disulfide) [60], have a significant impact on flavor. Esters, ketones, aldehydes, and carboxylic acids are mainly produced via the oxidation of free fatty acids generated via the hydrolyzation of lipids. LAB can regulate lipid metabolism and improve flavor [61].

#### 3.3.5. Mineral Composition 

**Figure 4:**
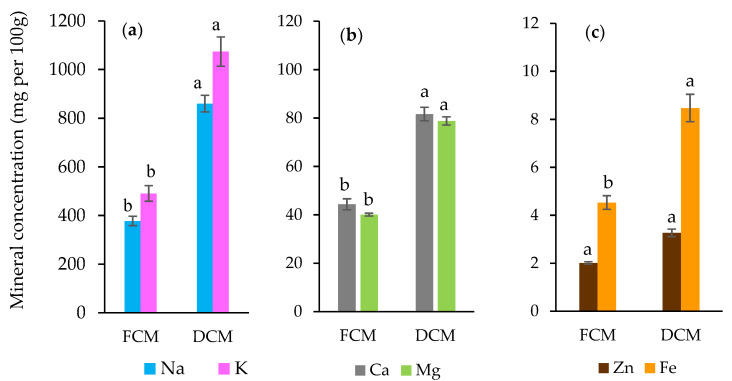
Mineral concentration of sodium and potassium (**a**), calcium and magnesium (**b**), and zinc and iron (**c**) in fresh (FCM) and dried camel merguez (DCM). Data are presented as the mean ± S.E. Different lowercase letters (a,b) indicate significant differences according to ANOVA (*p* < 0.05) between FCM and DCM.

The mineral compositions, including sodium, potassium, calcium, magnesium, iron, and zinc in mg per 100 g, are shown in Figure 4. All of the macro- and microelement components significantly increased (*p* < 0.05) and roughly doubled in quantity after drying. In particular, potassium for the macroelements (491 to 1070 mg per 100 g) and iron (Fe) for the microelements (4 to 8 mg per 100 g) were important, even in the FCM. Comparable trends for potassium and iron in camel meat composition were found [51,62]. The Fe concentration in camel meat is higher than in beef, mutton, and poultry [51]. Camels live in relatively extreme environments, which provide more dietary minerals than other herbivores’ typical range of habitats [63]. In addition to the meat, the coriander and fennel seeds used in merguez are reported as being rich in minerals, especially potassium, calcium, magnesium, and sodium macroelements [64].

## 4. Conclusions

This is the first study of authentic traditional dried camel merguez manufactured using traditional Tunisian methods. Dried camel merguez differs from other known dry-fermented sausages, containing a large quantity of spices and herbs (13.5%) and obtained using a simple sun-drying process. During drying, the weight loss reached 54%, giving a dried sausage characterized by low water activity (0.673), low moisture (12.3%), low pH (4.97), and good microbiological quality, allowing for storage for a long time at room temperature.

The composition of DCM includes several organic acids, mainly lactate (2820 mg/kg), giving evidence of important lactic acid fermentation despite the low lactic acid bacterial load of DCM (<10^4^). It contains diverse unsaturated fatty acids, in particular, oleic acid (33.1%), and various minerals, specifically iron (8mg per 100g), which promote the nutritional value of this traditional food. In addition, volatile compounds are present, with a high volume of terpenes (56.3%) contributing to its specific flavor.

The simulation of uncontrolled drying conditions should be studied to reproduce a safer and standardized product with the same flavor and bioactive components.

## Figures and Tables

**Table 1:** Fresh camel merguez formulation.

Ingredients	Composition (%)
Camel meat (biceps femoris muscles)	69
Hump fat	14
Ground hot red chili pepper	4.0
Crushed fresh garlic	3.5
Harissa	3.5
Tabeul	1.5
Natural sheep casings (16–18 mm)	1.5
Salt	2.0
Powdered fennel seeds	0.60
Crushed dried mint	0.40

**Table 2:** Microbiological parameters of fresh and dried camel merguez.

Samples	FCM (log CFU·g^−1^)	DCM (log CFU·g^−1^)
Mesophilic aerobic bacteria	7.9 ± 0.1 ^a^	5.2 ± 0.5 ^b^
Lactic acid bacteria	6.7 ± 0.05 ^a^	3.7 ± 0.08 ^b^
Enterobacteria	4.8 ± 0.1 ^a^	<2 ^b^
Yeast and mold	4.4 ± 0.2 ^a^	2.6 ± 0.01 ^b^
*Escherichia coli* β glucuronidase+	3.4 ± 0.4 ^a^	<1 ^b^
Sulfate-reducing anaerobic bacteria	2.3 ± 0.06 ^a^	<1 ^b^
Positive coagulase staphylococcus	<2 ^a^	<2 ^a^
*Listeria monocytogenes* per 25 g	n.d.	n.d.

FCM, fresh merguez with camel meat; DCM, dried merguez with camel meat. Data are presented as means ± S.E. Different superscript letters in the rows (a,b) of each examined parameter indicate significant differences according to ANOVA (*p* < 0.05). n.d. = not detected.

**Table 3:** Characterization of fresh and dried camel meat merguez.

Components (g/100 g)	FCM	DCM
Moisture	60.5 ± 1.0 ^a^	12.3 ± 0.4 ^b^
Protein	22.6 ± 0.6 ^b^	29.3 ± 0.9 ^a^
Fat	21.5 ± 0.2 ^b^	42.5 ± 0.3 ^a^

FCM, fresh merguez with camel meat; DCM, dried merguez with camel meat. Data are presented as the means ± S.E. Different letters in the same row indicate significant differences according to ANOVA (*p* < 0.05).

**Table 4:** Profile fatty acid (g/100 g of total fatty acid) of hump fat and dried camel merguez.

	Hump Fat	DCM
**Saturated Fatty Acids**		
Caprilic acid C8:0	0.0230 ± 0.0010	0.0230 ± 0.0010
Capric acid C10:0	0.0740 ± 0.0020	0.0630 ± 0.0010
Lauric acid C12:0	0.287 ± 0.002	0.267 ± 0.013
Tridecanoic acid C13:0	n.d.	0.0520 ± 0.0010
Myristic acid C14:0	6.22 ± 0.02	5.91 ± 0.52
Pentadecanoic acid C15:0	0.890 ± 0.030	0.960 ± 0.080
Palmitic acid C16:0	24.0 ± 0.03	25.7 ± 5.1
Heptadecanoic acid C17:0	1.31 ± 0.04	1.04 ± 0.34
Stearic acid C18:0	27.7 ± 0.8	24.3± 3.5
**Unsaturated Fatty Acids**		
Miristoleic acid C14:1	0.150 ± 0.090	0.125± 0.000
Palmitoleic acid C16:1	2.82 ± 0.21	3.68 ± 0.01
Heptadecenoic acid C17:1	0.600 ± 0.050	0.810 ± 0.100
Oleic acid C18:1 (*n*-9)	32.6 ± 0.1	33.2 ± 0.4
Linoleic acid C18:2 (*n*-6)	2.23 ± 0.05	3.31 ± 0.20
Eicosenoic acid, C20:1	0.190 ± 0.120	0.270 ± 0.001
Arachidonic acid C20:4 (*n*-6)	0.109 ± 0.001	n.d.

Data are means ± S.E., n.d. not detected.

## Data Availability

The original contributions presented in the study are included in the article, further inquiries can be directed to the corresponding author.
